# The Relationship Between the Healthy Diet Index, Chronic Diseases, Obesity and Lifestyle Risk Factors Among Adults in Kaunas City, Lithuania

**DOI:** 10.3389/fnut.2021.599567

**Published:** 2021-03-05

**Authors:** Audrius Dėdelė, Žydrūnė Bartkutė, Yevheniia Chebotarova, Auksė Miškinytė

**Affiliations:** Department of Environmental Sciences, Faculty of Natural Sciences, Vytautas Magnus University, Kaunas, Lithuania

**Keywords:** healthy diet index, food intake, obesity, chronic diseases, lifestyle risk factors

## Abstract

A healthy and balanced diet is an important factor contributing to overall health and wellness. The aim of this study was to develop a Healthy Diet Index (HDI) score and assess its association with various chronic diseases and lifestyle risk factors. A cross-sectional survey included 1,111 adults aged 18 years and older. Information on dietary habits was collected using a questionnaire. Additional demographic, socioeconomic and lifestyle risk factors data were also collected. Sixteen food groups were used to develop the HDI score for the residents of Kaunas city, Lithuania based on the national recommendations, World Health Organization (WHO) and other guidance on a healthy diet. We used logistic regression models to assess the association of the HDI score with chronic diseases, obesity and lifestyle risk factors. We found that both males and females were lacking the optimal consumption of the base components of a healthy diet–fruits and vegetables, starchy carbohydrates, and proteins. We also observed significant associations between the HDI score and several outcomes such as hypertension, arrhythmia, physical activity, and obesity. The suggested HDI score could serve as a valuable tool in assessing and improving dietary habits beneficial for promoting health and preventing many diseases.

## Introduction

Dietary risks were responsible for 7.94 million deaths and 188 million disability-adjusted life years globally among adults in 2019 (1). It was the third leading risk factor for attributable deaths. This aggregate risk factor was for all dietary risks: a diet low in whole grains, fruit, fiber, legumes, nuts and seeds, omega-3 fatty acids, polyunsaturated fatty acids, vegetables, milk, and calcium; and also diet high in sodium, trans fats, red or processed meat, sugar-sweetened beverages.

Eurostat findings show that both in 2016 and 2017, circulatory system diseases related to high blood pressure, cholesterol, diabetes and smoking were the leading causes of death in the European Union (EU−27) (2). Lithuania–along with Latvia, Hungary, and Slovakia–had the highest standardized rates of death from ischemic heart disease (heart attack), reporting 561, 397, 367, and 359 deaths per 100,000 inhabitants, respectively, in 2016. Lithuania also had the highest deaths per 100,000 inhabitants from a heart attack−536–compared to the rest of the EU-27 countries in 2017 (2).

The growing concern to create a simple, yet effective way to measure one's adherence to a healthy dietary pattern and its status has become one of the most important tasks to solve. Finding and following optimal healthy dietary plans could not only improve health status, but also lower the risk of non-communicable diseases (NCDs).

Different diet quality indices have been extensively researched by scientists taking into the consideration diet as a whole complex pattern, rather than concentrating on single foods or nutrients (3, 4). The associations between diet quality indexes and different health outcomes or nutrient adequacy were often being analyzed (5) and exposed high-quality diets as significantly lowering the risk of (NCDs) (6, 7). These researches are considered “*a priori,”* because they are based on dietary guidelines as opposed to “*a posteriori,”* which use statistical methods to analyze dietary patterns (4, 5, 8). Diet quality indices, such as Mediterranean Diet Score (MDS), Diet Quality Index-International (DQI-I) and the Dietary Approaches to Stop Hypertension (DASH) score were inversely associated with chronic disease risk markers (9); higher Healthy Eating Index- 2010 (HEI-2010), the alternative HEI-2010 (AHEI-2010), the Alternate Mediterranean diet (aMED) and DASH scores were associated with lower diabetes-related markers (10). Schwingshackl et al. (6) analyzed prospective cohort studies and revealed, that highest scores of HEI, AHEI, and DASH were associated with a significant reduction in the risk of all-cause mortality, cardiovascular disease (CVD), cancer, type 2 diabetes and neurodegenerative disease by 22, 22, 16, 18, and 15%, respectively, and inversely associated with overall mortality among cancer survivors by 12%. The MDS, the Mediterranean Adequacy Index (MDI) and the Healthy Diet Indicator (HDI) were inversely associated with all-cause mortality in the European elderly (11).

Lithuania has national, government-endorsed food-based dietary guidelines (FBDG) that were officially introduced by the Ministry of Health of The Republic of Lithuania in 2010 (12). Specific FBDG differ from country to country based on the availability, cultural acceptance, traditions of foods and the existing diet-related public health issues. Its main objects are providing general consumer education on the consumption of foods; ensuring the coverage or modification of nutrient needs or imbalances; reducing the risk of hypertension, hyperlipidemia, obesity, cancer, diabetes mellitus, osteoporosis, dental disease and CVD (13), which is especially important in Lithuania's context, as well as promoting overall well-being.

The principal aim of this study was to develop a Healthy Diet Index (HDI) score that could show adherence to the healthy diet recommendations. The secondary aim was to evaluate how the HDI score was associated with chronic diseases, obesity, and lifestyle risk factors among residents of Kaunas city, Lithuania.

Our study was cross-sectional, included a representative sample of the adult respondents of 18 and older, and analyzed their adherence to the healthy diet guidelines as well as the relationships of this adherence to chronic diseases, obesity and lifestyle risk factors. The present study will contribute to implementing a dietary scoring system for adults and analyzing its associations with various lifestyle risk factors, obesity, and chronic diseases in Lithuania and the Baltic countries as there is a lack of such studies in these regions. This study created a beneficial “*a priori*” tool that could be easily implemented in evaluating one's dietary habits and assisting in following the optimal healthy diet patterns.

## Materials and Methods

### Study Design and Sample

We conducted a cross-sectional survey, which was carried out by a well-acquainted with similar surveys market research agency via a telephone interview. This survey was conducted in the autumn of 2017 in Kaunas city, the second-largest city in Lithuania with 290,289 inhabitants (on July 1, 2017) located in the central part of the country. Single-stage telephone interviews included randomly selected 18-year-old or older adults residing in Kaunas city to represent the entire population of this city. A total of 1,111 respondents completed the survey. The response rate for the survey was 57%. Various demographic (age, gender, marital status, children), socioeconomic (educational level, employment, income), lifestyle and behavioral risk factors (smoking, alcohol consumption, perceived stress, physical activity (PA) and sedentary behavior) data were collected. Four age categories were set: (1) 18–30, (2) 31–45, (3) 46–60, and (4) 61 years and over. The education level was categorized into three groups: (1) low (secondary education), (2) medium (post-secondary education) and (3) high (university or college degree). According to the employment status, the participants were classified as employed or unemployed. Retirees, homemakers and students were assigned to the group of unemployed people. Unemployment rate, excluding retirees, homemakers and students, was 9.2% in our study. Three income (in Euro) categories were set based on the data of the monthly average household income by the Lithuanian Department of Statistics (Statistics Lithuania): (1) <1,000, 2) 1,000–1,500, and (3) >1,500. The body mass index (BMI) was calculated as weight (kilograms) divided by height (meters) squared and was classified into three categories: (1) <25 kg/m^2^ (normal weight), (2) 25–29 kg/m^2^ (overweight), and (3) ≥30 kg/m^2^ (obesity).

Adults were classified by parental status (living with minor children: “no,” “yes”). By the smoking status, the participants were classified into non-smokers and smokers. The participants were asked about alcohol use (“no” or “yes”). The participants were classified as consuming alcohol if they responded that they consume alcohol at least once a week or more. The levels of PA were classified into two categories according to the WHO recommended levels of PA for adults: (1) those who engaged in <150 min of moderate-intensity PA per week and (2) those who engaged in sufficient physical activity (sPA) and achieve at least 150 or more min of moderate-intensity PA per week. To assess sedentary behavior, the respondents were asked about the time they spent sitting on a usual weekday and weekend day, not including the time spent sitting at work. The study received ethical approval from the Kaunas Regional Biomedical Research Ethics Committee (Approval No. BE-2-16).

### Diet Evaluation Components

Based on the Lithuanian FBDG (12), 16 components (food groups) were used to develop the HDI score. Those 16 components were divided into 10 separate groups by recommended frequencies of the consumption within a week: (1) vegetables; (2) fruits; (3) cereals, pasta, rice; (4) dairy; (5) white meat, legumes, nuts; (6) fish and seafood, eggs; (7) potatoes (8) red meat; (9) butter, margarine and (10) junk food. Five options for the eating frequency were established: “daily;” “5–6 times a week;” “2–4 times a week;” “1 time a week,” and “never or rarely.”

### Scoring System and Tertiles

Each participant was interviewed and personally evaluated the frequencies of dietary habits. To understand an individual's dietary pattern, each component of the diet was assigned a score according to the recommended level of consumption. Frequencies of food intake are presented in Table 1. The summation of the numbers assigned to each food item (sixteen components in total) resulted in the HDI score. The frequencies of eating were dichotomized as “optimal” or “non-optimal” levels. The theoretical sums ranged from 16 (i.e., one did not reach any “optimal” level) to 80 (the maximum score). Higher values of the index indicated the highest odds to fulfill the healthy diet pattern.

**Table 1:** The frequency of dietary intake and scoring system.

**Food category**	**Frequency**	**Score**	**Dichotomized**
Vegetables	Daily	5	Optimal
	5–6 times a week	4	Non-optimal
	2–4 times a week	3	
	1 time a week	2	
	Never/rarely	1	
Fruits	Daily	5	Optimal
	5–6 times a week	4	Non-optimal
	2–4 times a week	3	
	1 time a week	2	
	Never/rarely	1	
Cereals Pasta Rice	Daily	5	Optimal
	5–6 times a week	4	Non-optimal
	2–4 times a week	3	
	1 time a week	2	
	Never/rarely	1	
Dairy	Daily	5	Optimal
	5–6 times a week	4	
	2–4 times a week	3	Non-optimal
	1 time a week	2	
	Never/rarely	1	
White meat Legumes Nuts	Daily	5	Optimal
	5–6 times a week	4	
	2–4 times a week	3	Non-optimal
	1 time a week	2	
	Never/rarely	1	
Fish and seafood Eggs	2–4 times a week	5	Optimal
	1 time a week	4	Non-optimal
	5–6 times a week	3	
	Daily	2	
	Never/rarely	1	
Potatoes	2–4 times a week	5	Optimal
	1 time a week	4	
	5–6 times a week	3	Non-optimal
	Daily	2	
	Never/rarely	1	
Red meat	1 time a week	5	Optimal
	Never/rarely	4	
	2–4 times a week	3	Non-optimal
	5–6 times a week	2	
	Daily	1	
Butter Margarine	1 time a week	5	Optimal
	Never/rarely	4	
	2–4 times a week	3	Non-optimal
	5–6 times a week	2	
	Daily	1	
Junk food	Never/rarely	5	Optimal
	1 time a week	4	Non-optimal
	2–4 times a week	3	
	5–6 times a week	2	
	Daily	1	

Different dietary assessment systems, based on the scoring of points, where diet quality increases with the score, are usually the ones analyzed (5, 8, 11, 14–16). Categorizing each frequency of consumption as “optimal” or “non-optimal” was based on the Lithuanian FBDG (12), the Lithuanian Healthy and Sustainable Nutrition Recommendations (HSNR) (17), WHO (18), FBDG in Europe (19) and other scientific literature:

1) Vegetables, fruits, cereals, pasta, rice - only “daily” frequency of consumption was assigned as “optimal” to these food groups as these foods are the key components of the healthy diet and should be consumed several times a day as they are a great source of energy, dietary fiber, microelements and vitamins (12, 17, 18). All of the remaining frequencies of consumption of these groups were considered as “non-optimal.”
2) Dairy, white meat, legumes and nuts–“optimal” consumption of these protein-rich foods was considered “daily” and “5–6 times a week.” According to the guidelines, red and processed meat should be replaced by white lean meat or other non-animal proteins. Consumption of legumes and nuts should be encouraged, replacing and not integrating animal foods (20). Minimum of 30 g/day of pulses, nuts and seeds are recommended by WHO Study Group of NCDs (21).
3) Fish and seafood, Eggs—“optimal” consumption was considered as only “2–4 times a week”. According to the Lithuanian HSNR (17), fish should be consumed 2–3 times a week (total of 300–450 g). At least 200 g should be oily fish—salmon, trout, mackerel, herring and also canned tuna. The same guidelines are also endorsed by Norway (22). Separate guidelines for seafood were not included, but due to traditional classification, it was equal to fish consumption and addressed accordingly. Many studies are debating egg consumption factor concerning the risk of CVD, nevertheless, it is assumed that older people or those suffering from hyperlipidemia should reconsider daily consumption of eggs (23). High cardiovascular risk participants who consumed 2–4 eggs per week had no increased CVD risk (24). No more than four yolks per week are also suggested by Dietary Approaches to Stop Hypertension (DASH) diet (25). Since the Lithuanian FBDG does not provide specific quantities for egg consumption a week, we decided to follow other EU countries (19) that had defined this frequency, and chose “optimal” consumption as 2–4 times per week (e.g., Belgium (Flanders region), Ireland—no more than seven, Greece, Romania—up to 4, Spain−4–5, Croatia−3–4, Italy, Malta−2–4, Cyprus, the Netherlands, Austria, Finland—up to three eggs per week).
4) Potatoes (preferably with their skin on)—“optimal” consumption of these starchy vegetables was assumed as “2–4 times a week” and “one time a week”. Potatoes are usually included in “Cereals and cereal products” group by frequency of consumption, like root vegetables containing carbohydrates, which is criticized by some researchers (20). Nonetheless, starchy vegetables are excluded from daily 400 g of vegetables (17, 18), but recommended to consume a few times a day, although no specific quantities for consumption of potatoes are provided in the Lithuanian FBDG (12). Some EU countries advise on eating potatoes daily (Bulgaria, Denmark, Germany, Estonia, Latvia, Ireland, Cyprus, Portugal, but others prefer to limit the intake (Greece, Malta, Romania—≤3 servings, Italy−1–2 servings a week, Hungary—maximum every other day). We do believe that potatoes could pose some risk due to the high glycemic index, especially for diabetic patients (26) and particularly fried, roasted with oil or fat, consumed with additional sauces of saturated fat and salt. However, potatoes cooked using healthy cooking methods over frying or roasting with oil or fat, should be consumed instead of refined pasta or rice. One portion per day of steamed or baked with skin potatoes without the addition of excess saturated fat, sugar or sodium was proven to result in better diet quality, K and fiber intake, without raising cardiometabolic risk, when consumed instead of refined grains (27). Chips, french fries or other processed potato products should be limited.
5) Red meat, butter and margarine—“optimal” consumption of these foods was categorized as “never/rarely” and “1 time a week.” It is following the Lithuanian FBDG (12), HSNR (17) and WHO (28). Red meat should be limited to <500 g a week due to associations with an increased risk of cancer (29). A daily intake of saturated fats should be <10 % of total fat intake (30%) (12, 18). Diet of an excessive intake of saturated fats could pose a risk for obesity and atherosclerosis (12), and saturated fats should be replaced by unsaturated ones.
6) Junk food—“optimal” consumption of this category was only “never/rarely,” as these products are highly processed and energy-dense, but poor in nutrients (12, 28).

After calculating the sum of the HDI score for each participant, the rate was divided into tertiles: the 1st tertile was >54; the 2nd tertile was 50–53; and the 3rd tertile was <49. The first tertile (as reference to compare the results) represented those who achieved a higher index and were considered to have a healthy diet. The second tertile outlined a moderately healthy intake. The participants who were in the third tertile did not meet the recommendations and were considered to have a weak diet.

### Chronic Diseases, Obesity, and Lifestyle Risk Factors

The research interviewers collected information about chronic diseases, stress, PA and obesity. The participants were asked if they have ever been diagnosed by a doctor or health professional with any chronic disease (“no” or “yes”) and then to specify the chronic disease. Chronic diseases included having at least one or more of the following: hypertension, arrhythmia, angina pectoris, diabetes, allergies, cancer, kidney disease. Hypertension and arrhythmia were selected for further statistical analysis due to being the most frequently named by the respondents out of all collected chronic diseases. Hypertension and arrhythmia are strongly associated with dietary and other lifestyle factors (30–33) and thus can influence the occurrence of CVD (34, 35).

Perceived stress was assessed by a questionnaire based on the commonly used and validated Reeder stress scale (36, 37). Seven statements about everyday stressful situations were used to evaluate perceived stress levels in the study participants (38). The subjects had to indicate to what extent each statement applied to them: exactly, to some extent, not very accurately, or not at all. The responses were scored from one (strongly agree) to four (strongly disagree), and the overall score was calculated by summing up the scores of all seven items. A summary score ranged from 7 (high perceived stress) to 28 (low perceived stress). The perceived stress variable was dichotomized into two categories by the median value: (1) <21 (high perceived stress), and (2) ≥21 (low perceived stress) to perform logistic regression analysis.

The level of PA was divided into two categories: individuals who engage in low levels of PA (<150 min/week) and those who set about in high levels of PA (at least 150 or more minutes/week).

Obesity was categorized based on BMI. According to the WHO, a BMI greater than or equal to 30 kg/m^2^ is classified as obesity. People with a BMI <30 kg/m^2^ were classified as non-obese and those with a BMI ≥30 kg/m^2^ were classified as obese.

### Statistical Analysis

Basic descriptive statistics of the study participants were presented as frequencies (N) and percentages (%). The descriptive measures–mean and standard deviation (SD)–were calculated. Associations between categorical variables were examined using the Pearson Chi-square test (two-sided).

To analyze the associations between the HDI score, chronic diseases, obesity and lifestyle risk factors, the odds ratios (ORs) were calculated with 95% confidence intervals (95% CI). Logistic regression was applied to distinguish those associations. All models were adjusted for the selected set of covariates including age (continuous), sex, marital status, education level, employment status, income, BMI (continuous), stress, smoking, alcohol consumption, chronic diseases, sedentary behavior (continuous), minor children and sPA. For all our analyses, the level of statistical significance was set at *p* < 0.05.

Statistical analysis was calculated using SPSS software version 26.0 (IBM Corp. Released 2019. IBM SPSS Statistics for Windows, Version 26.0. Armonk, NY: IBM Corp.).

## Results

Baseline characteristics of the study participants are presented in Table 2. The study included 1,111 participants of whom 57.7% were female and 42.3% were male. The mean age of the participants was 48.4 years (SD = 16.8); 34.9% of the participants had a high level of education, and 59.5% were employed. The mean BMI was 26.4 kg/m^2^ (SD = 4.7), and 59.9% of the participants were overweight or obese. The prevalence of smoking and alcohol consumption was 30.0 and 51.4%, respectively. Only 10.4% of the study participants achieved the recommended levels of PA for adults developed by the WHO.

**Table 2:** Baseline characteristics of the study participants (*n* = 1111).

**Variable**	***N* (%)**
Gender	
Women	641 (57.7)
Men	470 (42.3)
Age group, years	
18–30	205 (18.5)
31–45	323 (29.1)
46–60	286 (25.7)
≥61	297 (26.7)
Age, mean (SD)	48.4 (16.8)
Marital status	
Married	653 (58.8)
Divorced	131 (11.8)
Single	196 (17.6)
Widowed	131 (11.8)
Educational level	
Low	416 (37.4)
Medium	307 (27.6)
High	388 (34.9)
Employment status	
Employed	661 (59.5)
Unemployed	450 (40.5)
Income, Eur	
<1,000	545 (62.1)
1,000–1500	200 (22.8)
>1,500	132 (15.1)
BMI, kg/m^2^	
<25	362 (40.1)
25–29	388 (43.0)
≥30	153 (16.9)
BMI, mean (SD)	26.4 (4.7)
Minor children	
No	772 (69.5)
Yes	339 (30.5)
Smoking	
No	778 (70.0)
Yes	333 (30.0)
Alcohol	
No	540 (48.6)
Yes	571 (51.4)
Physical activity, min/week	
<150	996 (89.6)
≥150	115 (10.4)
Chronic diseases	
No	713 (64.2)
Yes	398 (35.8)
Hypertension	
No	868 (78.1)
Yes	243 (21.9)
Arrhythmia	
No	998 (89.8)
Yes	113 (10.2)

The classification and the intake of food groups by sex are shown in Table 3. A non-optimal consumption of vegetables, fruits and nuts was significantly prevalent in both sexes. However, women, compared to men, were more likely to consume optimal levels of vegetables, fruits and nuts. The prevalence of non-optimal consumptions of red meat (from “daily” to “2–4 times a week” intake according to the healthy diet recommendations) was significantly higher in males. In spread category, a significantly higher optimal intake of margarine observed among both sexes; nonetheless, men preferred a non-optimal butter consumption compared to women. Simultaneously, a sub-optimal intake of junk food was more frequently observed in males. Overall, the results show that the sample among both sexes is prone to non-optimal consumption of the base part of a healthy diet–fruits and vegetables, starchy carbohydrates and proteins. However, women were more likely than men to report frequent optimal food intake levels while avoiding junk food.

**Table 3:** The intake of food groups according to the recommended level of consumption by sex.

**Food group**	**Sex**		*p*-value^*^
	**Male *N* (%)**	**Female *N* (%)**	
Vegetables			**<0.001**
non-optimal	405 (86.2)	471 (73.5)	
optimal	65 (13.8)	170 (26.5)	
Fruit			**<0.001**
non-optimal	422 (89.8)	516 (80.5)	
optimal	48 (10.2)	125 (19.5)	
Legumes			0.366
non-optimal	410 (87.2)	547 (85.3)	
optimal	60 (12.8)	94 (14.7)	
Cereals			0.132
non-optimal	313 (66.6)	454 (70.8)	
optimal	157 (33.4)	187 (29.2)	
Potatoes			0.374
non-optimal	115 (24.5)	172 (26.8)	
optimal	355 (75.5)	469 (73.2)	
Rice			0.783
non-optimal	467 (99.4)	636 (99.2)	
optimal	3 (0.6)	5 (0.8)	
Pasta			0.702
non-optimal	467 (99.4)	638 (99.5)	
optimal	3 (0.6)	3 (0.5)	
White meat			0.662
non-optimal	431 (91.7)	583 (91.0)	
optimal	39 (8.3)	58 (9.0)	
Red meat			**<0.001**
non-optimal	293 (62.3)	229 (35.7)	
optimal	177 (37.7)	412 (64.3)	
Fish and seafood			0.621
non-optimal	376 (80.0)	505 (78.8)	
optimal	94 (20.0)	136 (21.2)	
Dairy products			0.495
non-optimal	315 (67.0)	417 (65.1)	
optimal	155 (33.0)	224 (34.9)	
Eggs			0.117
non-optimal	281 (59.8)	353 (55.1)	
optimal	189 (40.2)	288 (44.9)	
Nuts			**0.037**
non-optimal	452 (96.2)	598 (93.3)	
optimal	18 (3.8)	43 (6.7)	
Butter			**0.008**
non-optimal	277 (58.9)	326 (50.9)	
optimal	193 (41.1)	315 (49.1)	
Margarine			**<0.001**
non-optimal	92 (19.6)	68 (10.6)	
optimal	378 (80.4)	573 (89.4)	
Junk food			**<0.001**
non-optimal	181 (38.5)	116 (18.1)	
optimal	289 (61.5)	525 (81.9)	

* *p-values were calculated from the Chi-square test. Values in bold indicate significance at the p < 0.05 level*.

We have developed the HDI score described above and evaluated its association with chronic diseases and lifestyle risk factors (Table 4). In the crude logistic regression models, individuals in the second HDI score tertile had increased odds of chronic disease, hypertension, arrhythmia, and obesity compared to those in the first tertile. Participants with the lowest HDI score (the 3rd tertile) had significantly higher odds of chronic disease (OR = 1.86, 95% CI 1.37–2.53), hypertension (OR = 3.03, 95% CI 2.04–4.50) arrhythmia (OR = 4.84, 95% CI 2.58–9.08), and obesity (OR = 2.30, 95% CI 1.40–3.79), and had reduced odds of reaching sPA levels (OR = 0.47, 95% CI 0.29–0.78).

**Table 4:** Crude and adjusted odds ratios (aOR) and 95% confidence intervals of chronic disease, hypertension, arrhythmia, stress, physical activity, and obesity according to the Healthy Diet Index (HDI) score.

**Outcome**	**Tertiles of Healthy Diet Index (HDI) score**					
	**Crude OR (95% CI)**			**aOR (95% CI)**		
	**1**	**2**	**3**	**1**	**2**	**3**
Chronic diseases^a^	1	1.54 (1.11–2.14)^*^	1.86 (1.37–2.53)^***^	1	1.09 (0.64–1.85)	0.98 (0.60–1.60)
Hypertension^b^	1	2.20 (1.44–3.37)^***^	3.03 (2.04–4.50)^***^	1	1.88 (1.03–3.41)^*^	2.15 (1.23–3.75)^**^
Arrhythmia^c^	1	2.59 (1.30–5.16)^**^	4.84 (2.58–9.08)^***^	1	1.24 (0.52–2.93)	2.49 (1.16–5.35)^*^
Stress^d^	1	0.87 (0.64–1.19)	1.12 (0.84–1.49)	1	0.95 (0.60–1.49)	1.26 (0.84–1.89)
Physical activity^e^	1	1.09 (0.69–1.71)	0.47 (0.29–0.78)^**^	1	0.86 (0.43–1.72)	0.27 (0.12–0.60)^**^
Obesity^f^	1	2.90 (1.73–4.88)^***^	2.30 (1.40–3.79)^**^	1	2.55 (1.47–4.44)^**^	1.65 (0.96–2.83)

* p < 0.05;

** p < 0.01;

*** *p < 0.001; a model adjusted for age, marital status, education level, employment status, BMI, stress, smoking, and sedentary behavior; b model adjusted for age, sex, marital status, education level, employment status, BMI, stress, smoking, and alcohol consumption; c model adjusted for age, sex, education level, employment status, BMI, stress, and alcohol consumption; d model adjusted for age, sex, employment status, income, chronic disease, BMI, smoking and, children's age; e model adjusted for age, sex, marital status, education level, employment status, BMI, smoking, and children's age; f model adjusted for age, sex, education level, sedentary behavior, chronic disease, alcohol consumption, smoking, children's age, and sPA*.

After adjustment for risk factors, data analysis revealed that the risk of obesity was significantly higher in the second tertile (aOR = 2.55, 95% CI 1.47–4.44) than in the third tertile showing a non-significant increase in risk. The results showed that having the lowest HDI score was significantly associated with more than a 2-fold higher risk of hypertension (aOR = 2.15, 95% CI 1.23–3.75), a 2.5-fold higher risk of arrhythmia (aOR = 2.49, 95% CI 1.16–5.35), and by 73% reduced odds of reaching sPA levels (aOR = 0.27, 95% CI 0.12–0.60), compared to individuals with the highest HDI score (the 1st tertile). However, the effect of the HDI score on the risk of chronic disease and stress was not statistically significant.

## Discussion

The principal aim of the present study was to develop an HDI score for the residents of Kaunas city, Lithuania and to explore the associations between the HDI score, chronic diseases, obesity and lifestyle risk factors. Our study examined adherence to healthy diet recommendations, and we strongly believe the developed system will be beneficial in reorganizing and enhancing public nutrition patterns.

Our research showed that although both males and females were not consuming optimal levels of suggested main food groups of the healthy diet (vegetables, fruits), males were found to consume the least preferential food groups (red meat and junk food) more frequently, compared to females. These tendencies were also observed in other nutrition habits of adults and elderly surveys, performed in Lithuania (39, 40) and also in nutrition and nutritional habits evaluation of Vilnius city dwellers in 2007 (41), which both testified, that Lithuanians did not consume sufficient amounts of vegetables and fruits, although significantly more women than men consumed dairy products, vegetables and fruits; while men, compared to women, were more likely to consume more meat high in fat. Nonetheless, overall nutrition habits of Lithuanians have become healthier: 1994–2010 national surveys have demonstrated, that the proportion of persons spreading butter on bread has decreased and the consumption of fresh vegetables and vegetable oil in cooking has increased (42).

The most significant associations were found between weak diet and hypertension, arrhythmia, PA and obesity. It remained significant after controlling for potential confounders. Adopting beneficial dietary patterns rich in nutrients, unsaturated oils, low-fat dairy products, and lean protein while limiting added sugars, salt, saturated fatty acids, and refined carbohydrates have been proven to reduce CVD risk (43, 44). It is especially important to improve dietary patterns for hypertensive patients before taking antihypertensive medications. Stage 1 hypertension (systolic blood pressure 140–159 mmHg/diastolic blood pressure 100–109 mmHg) can be positively modified with the healthy diet approach. A reduction may vary from ~ −5.5 for systolic blood pressure and −3.0 mmHg for diastolic blood pressure (45), by −7.1 mmHg and −2.6 mmHg, respectively (46), or even by −10–11 mmHg and −7–8 mmHg, respectively (47), compared to the low-fat diet. Mediterranean and DASH (Dietary Approaches to Stop Hypertension) diets should be the ones to follow and are usually recommended by healthcare providers for CVD risk control (8, 43, 44, 48).

Residents following a weak diet had the biggest risk of arrhythmia, compared to those leading a healthy diet. According to scientists, cardiac arrhythmia—atrial fibrillation—was linked to alcohol abuse, the intake of fish-derived n-3 polyunsaturated fatty acids and coffee consumption (49). Consumption of ≥3 portions of nuts a week was associated with a lower risk of atrial fibrillation, compared with non-consumers (50). The Mediterranean diet with extra-virgin olive oil (50 g or four tablespoons a day) was proven to reduce the risk of atrial fibrillation, compared to the low-fat diet (51). Recommendation to replace animal fats by olive and rapeseed oils is advised in the Lithuanian FBDG (12), although more emphasis should be placed on the preferred quality of these oils.

Another significant inverse association was found between weak diet and PA. Respondents with weak dietary habits were the ones to be the least physically active and not achieving the 150 min/week of PA recommended by the WHO. This association was also found to be the same in other studies (8, 48, 52, 53) where higher adherence to a healthy diet was associated with higher PA. Children and adolescents with higher levels of PA were found to consume more healthy foods and to like juice, water, milk, dairy products, fruit, and vegetables compared to those with lower PA levels (54). A cross-sectional study carried by Yang et al. (55) found that participants with a lower BMI and higher PA levels had lower scores in fast food consumption. Atkins et al. (56) performed a prospective study of CVD in 3,226 men aged 60–79 years, which showed that adherence to the “high-fat/low-fiber” diet was positively associated with not only current smoking, heavy drinking, and obesity, but also with physical inactivity. Another study (57) highlighted that PA perception programs should include a nutrition component as a strong significant factor in improving metabolic, physical, and psychological health.

Respondents in the second HDI tertile (moderately healthy intake) had the highest odds of being obese, compared to the respondents in the first HDI tertile (healthy diet). Therefore, we cannot declare that the lowest HDI score was consequently associated with the highest odds of obesity. Although in other studies the lowest adherence to healthy eating indices was associated with the biggest risk of obesity (8, 52, 55), Guo et al. suggested that high HEI score may not be particularly associated with obesity, especially considering the fact the HEI are not specifically targeting the obesity (58); our survey was also considering the importance of consuming optimal frequencies of various, nutritious and beneficial to overall health and well-being food groups, preferring plant-based foods over animal-based foods (12, 17).

Having been diagnosed with chronic diseases was also related to a weak diet in the univariate analysis; however, this association became non-significant after adjusting for the covariates. Similar results were also obtained by other researchers (53), who showed that dietary intakes could result in different health outcomes when other covariates were involved. This indicates that a holistic approach should be taken when considering the prevention of different health risk factors due to interaction between each of them, which creates an even more significant synergistic risk. Adjusting or modifying one of the interacting risk factors may present a valuable positive effect on the overall health status and well-being.

The HDI score we developed was used to measure the extent to which dietary intake complied with official dietary recommendations. Questions on the frequency of consumption of specific 16 food groups were addressed using five frequency categories ranging from “never or rarely” to “daily.” Other dietary surveys use food frequency questionnaires (daily, weekly or monthly) (3). A Baltic Sea Diet Score (BSDS), which evaluated the adherence to the healthy Nordic diet in the Finish population, used a 131-item FFQ with nine frequency categories from ranging from “never or seldom” to “at least six times a day” (59). The BSDS contained ten food groups and included saturated fatty acids, polyunsaturated fatty acids, carbohydrates, sucrose, protein, alcohol, and fiber. The resulting score ranged from 2 to 25, while the HDI score we developed ranged from 16 to 80.

For example, the Healthy Eating Index (HEI) consisted of 10 components (grains, vegetables, fruits, meat, milk, fat energy, saturated fat energy, cholesterol, sodium and variety in diet) with the score ranging from 0 to 100 (3, 7).

This HDI score we created consisted of food group indicators only, the same practice was used for many other indices–for example, the Dietary Behavior Score (DBS)-2009, the Dietary Quality Score (DQS)-2007, Modified Mediterranean Diet Score (mMDS)-2014 (60), to name a few, although the original Healthy Diet Indicator (HDI) score consisted of both food groups and nutrients (5). Our HDI score did not distinguish between whole and refined grains, which was the same approach used for the Dietary Quality Index (DQI), Healthy Eating Index (HEI), Mediterranean Diet Score (MDS) and Healthy Diet Indicator (HDI) score (5).

This study has made strong contributions. We acknowledged the importance of following and analyzing adherence to the healthy diet pattern. The associations between the HDI score and various factors were calculated with adjustment for socio-economic, demographic, and other lifestyle and behavioral covariates. We created a practical and beneficial “a priori” HDI score tool that could easily be implemented by the interested parties. The adult healthy eating scoring system and its associations with different factors have not been analyzed in the Baltic states thus far. The evaluation and monitoring of dietary patterns would be especially helpful for citizens with higher CVD and lifestyle risk factors.

The Lithuanian FBDG (12) includes a pyramid as a food guide graphical representation, which was composed in 2010. Some of the Lithuanian recommendations we discussed earlier need revisions, especially underlining the importance of “whole grains,” “red and processed meat” vs. “lean white meat/poultry,” providing more detailed explanations of quantities for daily consumption of all the illustrated groups. We would suggest updating and revising them in line with the latest nutrition recommendations and/or adopting good practices in providing thorough quantities of consumption as recommended by other EU countries. We are also campaigning to pay attention to the effects on the health of the various components in the food group. For example, give preference to whole grains and diversify the diet with multicolor vegetables and fruits multiple times on an everyday basis.

### Limitations

This study contains a few limitations. Overall adherence to the existing FBDG based on the 16 different food groups was investigated. The research was carried only in the urban population of one city; therefore, some degree of bias is expected. Another limitation of this study is a lower than expected response rate which may reduce the statistical power of the results of the study. However, a random selection of a sample population of the study allows to avoid bias in the results.

We analyzed food groups according to the existing Lithuanian FBDG (13) and healthy eating recommendations (17), but due to insufficient recommendations on quantities of some specific food groups, we had to research additional sources (18, 19).

Our questionnaire did not include separate entries for the frequency of consumption of specifically “whole grain” cereals and/or products, “processed meat,” “sweets.” These specific groups were attributed to the “cereals, pasta, rice,” “red meat” and “junk food” categories, respectively, by the respondents. Future enhancements of these study components should be implemented and reviewed.

Our survey did not take into account specific micro- and/or macro- nutrients, including sodium, as incorporated in other dietary quality indices (3, 15, 60), the relevance of the specific food groups was prioritized, which could also be adjusted in future studies.

Although food intake ratio/food frequency questionnaires are usually used in these types of studies (8, 53, 54), the respondents had to self-evaluate and/or recall the ratio of certain food groups consumed; the levels of perceived stress, PA and sedentary behavior were also self-reported. This could result in HDI score classification errors and could misrepresent the calculated associations between the HDI score, chronic diseases, obesity and lifestyle risk factors. For PA measurement, we used the WHO recommendation of achieving at least 150 or more minutes of moderate-intensity PA per week. Future study, accounting for vigorous-intensity PA of at least 75 min per week, could also be considered. Due to the nature of this study, it is quite complicated to determine if the HDI score affected other various researched outcomes or vice versa, as implicated in another similar study (53).

The questionnaire did not consider quantities of portions or servings consumed; frequencies of the analyzed food groups intake were the principles of the HDI score compilation. Future enhancements could be performed to strengthen the validity of the HDI score. The daily ratio of food intake journals, diaries, or photographs of the suggested serving or portion size could produce more accurate results.

## Conclusions

The proposed system is a simple tool for evaluating and measuring one's adherence to the healthy diet recommendations. A significant association was found among the participants in adjusted models where having the lowest HDI score was associated with a higher risk for hypertension, arrhythmia, obesity, and reduced odds of reaching sPA compared to the healthy diet model. The HDI score has the potential of serving as a useful tool considering additional improvements on specific food groups, including portion sizes and nutrients for the evaluation of the existing nutrition recommendations considering and their associations with chronic diseases and lifestyle risk factors.

## Data Availability Statement

The anonymized raw data supporting the conclusions of this article can be obtained upon reasonable request to the corresponding author.

## Ethics Statement

The studies involving human participants were reviewed and approved by Kaunas Regional Biomedical Research Ethics Committee (Approval No. BE-2-16). Written informed consent for participation was not required for this study in accordance with the national legislation and the institutional requirements.

## Author Contributions

AD and AM: conceptualization. AM and YC: methodology and software. YC: formal analysis and visualization. AD: data curation and supervision. ŽB and YC: writing—original draft preparation and writing—review and editing. AM: project administration. All authors: have read and agreed to the published version of the manuscript.

## Conflict of Interest

The authors declare that the research was conducted in the absence of any commercial or financial relationships that could be construed as a potential conflict of interest.
